# Supplementary material for: HIVprotI: an integrated web based platform for prediction and design of HIV proteins inhibitors
Source: J Cheminform. 2018 Mar 9;10:12. doi: 10.1186/s13321-018-0266-y (PMC5845081; doi:10.1186/s13321-018-0266-y)
Supplement: Supplementary file 2 — Additional file 2. Source code of HIVProtI web server. [file 13321_2018_266_MOESM2_ESM.zip › HIVprotI_Source-code/hivprotI/weka-3-6-6/doc/weka/clusterers/package-frame.html]

weka.clusterers


weka.clusterers

Interfaces 
  
*Clusterer*
  
*DensityBasedClusterer*
  
*NumberOfClustersRequestable*
  
*UpdateableClusterer*

Classes 
  
AbstractClusterer
  
AbstractDensityBasedClusterer
  
CheckClusterer
  
CLOPE
  
ClusterEvaluation
  
Cobweb
  
DBScan
  
EM
  
FarthestFirst
  
FilteredClusterer
  
HierarchicalClusterer
  
MakeDensityBasedClusterer
  
OPTICS
  
RandomizableClusterer
  
RandomizableDensityBasedClusterer
  
RandomizableSingleClustererEnhancer
  
sIB
  
SimpleKMeans
  
SingleClustererEnhancer
  
XMeans
